# Physiological responses and behavioural organization of very low birth weight infants during swaddled versus traditional weighing

**DOI:** 10.1002/nop2.1943

**Published:** 2023-07-17

**Authors:** Silvia Vicente‐Perez, Gemma Robleda, Ignasi Gich, Tania Nolla, Jaume Ponce‐Taylor, Sergio Verd, Gemma Ginovart

**Affiliations:** ^1^ Neonatal Intensive Care Unit Hospital de la Santa Creu i Sant Pau Barcelona Spain; ^2^ Campus docent Sant Joan de Déu Barcelona University Barcelona Spain; ^3^ Iberoamerican Cochrane Centre Hospital de la Santa Creu i Sant Pau Barcelona Spain; ^4^ Clinical Epidemiology Unit Hospital de la Santa Creu i Sant Pau Barcelona Spain; ^5^ Orthopedic & Neuroscience Units Hospital de la Santa Creu i Sant Pau Barcelona Spain; ^6^ Department of A&E Primary Care Balearic Health Authority Majorca Spain; ^7^ Department of Primary Care Balearic Health Authority, La Vileta surgery Majorca Spain; ^8^ Neonatal Intensive Care Unit Hospital Germans Trias Barcelona Spain

**Keywords:** behaviour, breastfeeding, clothing, neonate, nursing care, premature infant, vital signs, weights and measures

## Abstract

**Aim:**

Despite the consequences of neonatal distress and agitation, preterm infants undergo stress owing to weighing procedures. The objective of this study was to enable very low birth weight infants to maintain adequate self‐regulation during weighing.

**Design:**

This prospective crossover study utilizes a within‐subjects design, where intervention days were compared to control days.

**Method:**

Infants were exposed to both swaddled and unswaddled weighing in an intensive care nursery setting. Nineteen very low birth weight infants were weighed on two consecutive days. Variables of heart rate, respiratory rate and ALPS‐Neo score were recorded.

**Results:**

Stress score decreased significantly from 1.65 (pre‐weight) to 0.23 (weight measurement) in swaddled‐intervention periods; conversely, it increased significantly from 1.26 (pre‐weight) to 4.97 (weight measurement) in control periods. During weight measurement, heart and respiratory rate were significantly lower for swaddled‐intervention days when compared to control days. Given the significant impact of swaddled weighing in reducing stress, this method can be used as an appropriate weighing procedure in intensive care. This research has no patient or public contribution.

## 1 INTRODUCTION

Very low birth weight (VLBW) infants admitted to neonatal intensive care units (NICU) are exposed to parent‐infant separation, bright lights, excessive noise levels, painful interventions and other stressful stimuli. Agitation in neonatal life impacts on multiple regions of the developing brain (Hatfield et al., 2019), can adversely affect the maturation of vision and hearing, sleeping patterns, or growth, and consequently impair neurodevelopmental outcomes (McPherson et al., 2020). In the short‐term, distress and agitation have been reported to worsen caloric consumption, bradycardia and oxygen desaturation in newborn infants (Field, 2017; Taplak & Bayat, 2021). In the mid‐ to long‐term, untreated crying can lead to poor food intake, delayed wound healing, irritability and regression of the infant's development (Golianu et al., 2007; Imbulana et al., 2017). Greater exposure to pain in preterm infants predicts thinner cortex in the frontal and parietal lobes, as well as smaller volumes of the limbic system and basal ganglia (Casavant et al., 2019; McPherson et al., 2020; Schmitz‐Koep et al., 2021).

Therefore, interest in interventions to reduce the stress levels experienced by the VLBW infant has increased. Daily weighing is a vital component of routine neonatal care, but is still a stressful experience for VLBW infants. Decisions regarding fluid status, nutrition requirements and drug doses of each patient are derived from daily weight variations. Swaddling is a technique in which environmental adversity is minimized. Historically, when infants are swaddled, they cry less, maintain a more regular heart rate (HR) and exhibit less motor stress than when unswaddled in the NICU (Hatfield et al., 2019; Huang et al., 2022; Kommers et al., 2018; Lejeune et al., 2021). Enhanced interaction with the infant, facilitated parental attachment and decreased parental stress are additional benefits of keeping the swaddled newborn in a calm and alert state (Fern et al., 2002), but there is minimal research into the impact of swaddled weighing on premature infants. This study was conducted in order to compare the effects on stress‐related behaviours of a newly developed swaddling device with conventional weighing methods among VLBW infants.

## 2 METHODS

### 2.1 Setting

This is an analysis of VLBW infants from the NICU of Sant Pau Hospital, a tertiary referral centre in Barcelona, Spain. The unit consists of 10 intensive care and seven high‐dependency care cots, with around 350 admissions yearly and a bed occupancy rate of 90%.

### 2.2 Participants

All VLBW infants admitted to our 17‐bed, Level III NICU from September 2016 to October 2017 were asked to participate at the earliest appropriate occasion after admission. Infants were deemed eligible provided they were clinically stable, older than 1 week of life, and fed human milk. Exclusion criteria were as follows: haemodynamic instability, phototherapy, major surgery, sedation, formula feeding, severe brain pathology, major congenital defects or consumption of illicit substances by their mothers during pregnancy. Typically, in our hospital, all VLBW infants routinely receive human milk feeds.

### 2.3 Sample

Based on the expected respiratory rate (RR) mean value differences (51.8 vs. 41.5 breaths/min), derived from the only previous study on physiologic and behavioural distress of preterm infants during swaddled versus unswaddled weighing (Neu & Browne, 1997), a 19‐subject sample size (power of 0.8 and *α* = 0.05) was selected for the study. The same panel of VLBW infants was surveyed repeatedly over time. Twenty‐three eligible infants were admitted to the NICU during the data‐collection period. One infant was not fed human milk, one infant was under deep sedation and two sets of parents declined to participate. These four infants did not differ from the sample in illness severity or birth weight.

### 2.4 Design and intervention

This prospective crossover study utilizes a within‐subjects design, where intervention days were compared to control days. Participants were randomized into two groups (Group 1 and Group 2) to receive a different intervention on each of 2 days (Day 1 and Day 2). Half of the infants (Group 1) started with routine care (control days), while the other half started with swaddling care (Group 2). Group 1 babies were weighed according to the hospital standard procedures, without any intervention on Day 1; subsequently, a swaddling device was used during weighing care sessions on intervention day (Day 2). Group 1 infants were weighed conventionally on odd days (Day 1) and weighed swaddled in a not marketed swaddling device (*SWADDY*) on even days (Day 2). This sequence was applied inversely to babies in Group 2. Conditions for both groups were low‐intensity light, minimal noise, gentle movements during the weighing procedure, and no handling from 20 min before to 60 min after weighing. The swaddling device device was weighed in isolation, before each intervention, and has always weighed between 20 and 30 grams. During any period of evaluation, any unnecessary interventions were avoided.

**FIGURE 1:**
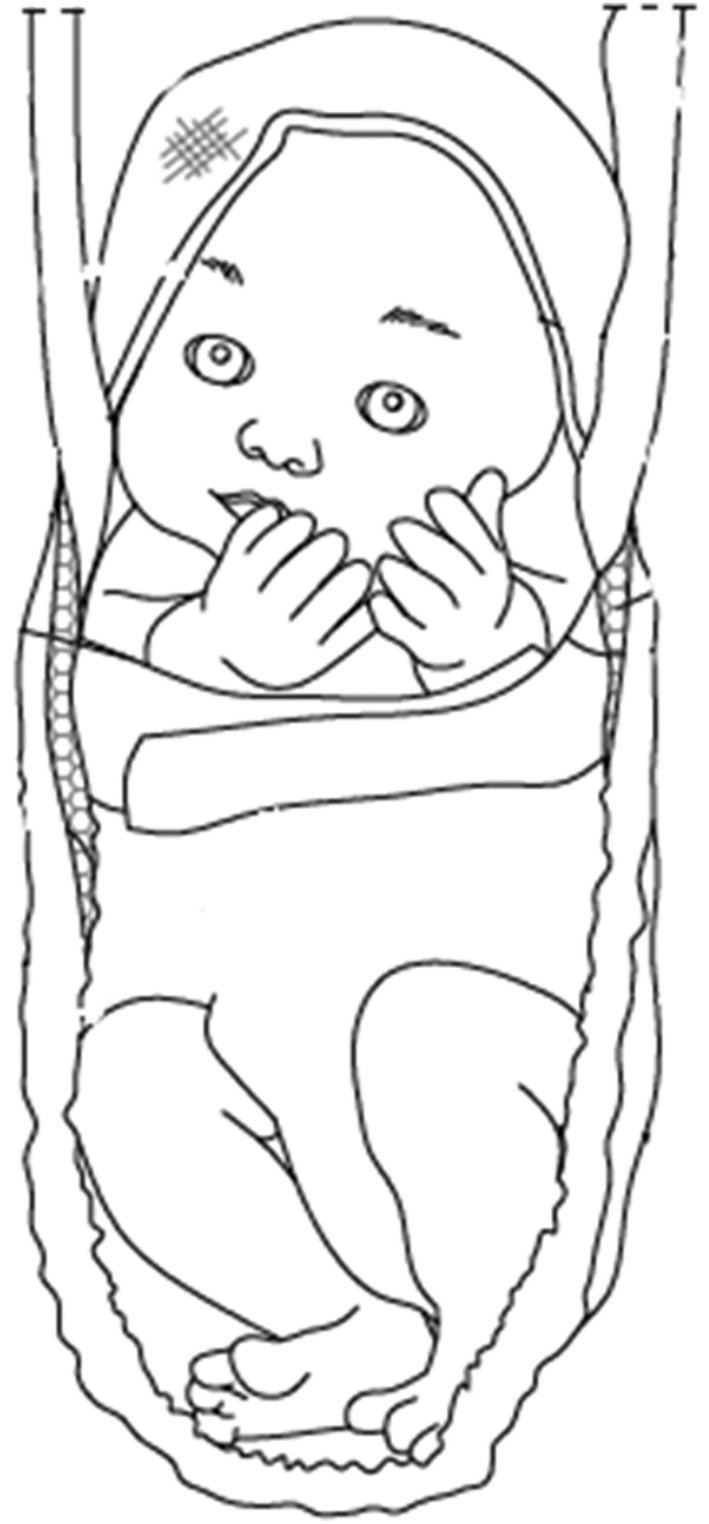
Swaddy. In the period of swaddling, a cotton cloth was wrapped around the infant so as to cover his/her upper trunk and somehow brings his/her arms closer to the midline. The baby lies with legs semi flexed to absorb any shocks.

In the period of swadding, the light‐coloured, soft, thin cloth (*SWADDY*) was wrapped around the infant so as to cover his/her upper trunk and limit his/her arms movements. The cap of the garment allows head‐trunk alignment, thereby ensuring that the airway remains unobstructed (Figure 1).

For our study, the step‐by‐step guide to swaddling an infant was adopted. Infants were placed in a supine position on a blanket while their arms were flexed, and close to the chest. The end of each side of the garment was crossed over the neonate's chest to the opposite side and tucked over the elbow, securing the arms of the infant in flexion and hands easily accessible to the mouth, to procure self‐regulation of the infant. To ensure that the device was not over‐restrictive, *SWADDY* was checked by sliding a finger between the wrap and the neonate. Feet were positioned in the lower portion of the garment. This was done to keep hips and knees flexed, and to reduce tension in abdominal muscles. Arms were wrapped tightly. From the hip downwards, the wrapping was less tight, allowing for leg abduction.

### 2.5 Data collection

Close monitoring and strict measurement of vital signs was part of the work of specialist nurses in the intensive care unit. Two research nurses, not routinely involved in neonatal care, analysed ALPS‐Neo score before, during, and after weighing care, with and without *SWADDY*. Although blinding of the observers was not possible, they were instructed to perform the measurements in the same environmental conditions for every newborn. All infants were monitored for heart rate and respiratory rate (HR, RR) using the continuous display on a General Electric MD15T monitor. HR and respirations were recorded five times every 4 min before the weighing to establish a baseline average for vital signs comparison. The nurses continued the physiologic data recording throughout the weighing procedure and additionally 60 min after the weighing to assess the degree of organization after this procedure.

ALPS‐Neo scale was scored 20 min before handling, immediately after weighing, and 60 min after weighing. ALPS‐Neo is a five‐item scale for the continuous evaluation of preterm infants' behavioural responses to pain and stress (Lundqvist et al., 2014). It includes scores for facial expression, breathing pattern, tone of extremities, hand and foot activity, and level of activity. Scores range from zero to two, with zero indicating a well‐organized functioning subsystem, and two indicating a very disorganized functioning subsystem. The total sum obtained from the scale items ranges from zero to ten. High scores signify a high stress level. A score below 3 means that the infant is able to maintain self‐regulation without nurse assistance, a score from 3 to 5 means that the infant needs individualized supportive care intervention, and a score above 5 means that the infant may need pharmacological intervention. Two independent observers measured behavioural responses using the ALPS‐Neo scale. The kappa inter‐observer coefficient was >0.6 for 70% of measurements, and <0.4 for 6% of measurements.

### 2.6 Data analysis

Demographic and clinical characteristics of the infants were analysed using frequencies for nominal level variables, means and standard deviations for continuous variables. The infants served as their own controls in this crossover design aimed at maximizing power. Differences from baseline to during and after weighing scores for the three dependent variables (HR, RR and APLS‐Neo scale) were examined by independent samples *t*‐test. Statistical significance was set at *p <* 0.05. Collected data were analysed using the IBM Statistical Package for the Social Sciences.

### 2.7 Ethics approval

This study was conducted with the approval of the University Hospital Clinical Research Ethics Committee. The committee gave its favourable opinion on 22 August 2016, which was registered under the name: IIBSP‐EST‐2015‐97. The authors certify that they have obtained all appropriate patient consent forms. In each case, a member of the nursing staff of the neonatal unit purposely contacted the parent of the newborn who was a candidate for participation in the study, explained orally the procedures and objectives of the study, and requested that, if the family felt it appropriate, they give their consent by signing the written document given to them containing all the details of the intervention.

## 3 RESULTS

### 3.1 Sample

We screened 23 preterm babies for eligibility, and 19 consecutive subjects were enrolled (14 males/5 females). The main reasons for exclusion were: withheld consent (10%), deep sedation (5%) and formula feeding (5%). The profile of the study subjects is shown in Table 1. For our sample of 19 VLBW infants (mean gestational age of 30.0 weeks), we analysed 190, 38, and 38 care sessions corresponding to pre‐weight, weight and post‐weight periods, respectively. Regarding ventilation, seven infants received no ventilation, and twelve infants (64%) received non‐invasive ventilation.

**TABLE 1:** Patient characteristics.

Number of patients	19
Gender	5 female/14 male
Gestational age, weeks (mean, SD)	30.05 (2.26)
Birth weight, g (mean, SD)	1908 (304)
Human milk/formula	19/0
Spontaneous breathing	7 (36.8%)
Continuous positive airway pressure	1 (5.2%)
*High*‐*flow* nasal *cannulae*	6 (31.5%)
*Nasal Bi‐level* positive airway pressure	5 (26.3%)
Number of pre‐weight sessions of care	190
Number of weight measurements	38
Number of post‐weight sessions of care	38

Abbreviations: g, gram; SD, standard deviation.

Table 2 shows comparative data between intervention‐days and control‐days. During weighing, the treatment differences were highly significant for all dependent variables. The mean HR and RR were 16% and 43% higher, respectively, during unswaddled weighing with respect to swaddled weighing, although within clinical normal limits. More notably, during weighing, the ALPS‐Neo score indicates need for supportive care intervention among unswaddled babies (mean 4.97), in contrast to adequate self‐regulation (mean 0.23) among swaddled babies. With regards to the pre‐weight care periods, RR was significantly lower in control days than in *SWADDY*‐days. Thereafter, post‐weighing RR and ALPS‐Neo scores were significantly lower in *SWADDY*‐days than in control‐days.

**TABLE 2:** Comparison between intervention‐days and control‐days. Mean value (standard deviation) of vital signs in the pre‐weight care, during weight measurement and in the post‐weight care periods.

	Pre‐weight care			During weight measurement			Post‐weight care		
Vital signs	Swaddy intervention	Control weighing	*p* Value	Swaddy intervention	Control weighing	*p* Value	Swaddy intervention	Control weighing	*p* Value
ALPS‐Neo score	1.65 (1.45)	1.26 (1.28)	0.380	0.23 (0.42)	4.97 (1.92)	**<0.0001**	0.05 (0.22)	1.65 (1.59)	**0.0001**
Respiratory pattern	0.29 (0.46)	0.39 (0.54)	0.308	0.13 (0.34)	0.89 (0.66)	**<0.0001**	0.03 (0.16)	0.16 (0.37)	0.068
Respiratory rate	58.10 (9.53)	46.91 (8.54)	**0.0005**	46,11 (14.10)	66.47 (11.49)	**<0.0001**	42.32 (9.38)	54.84 (11.57)	**0.0008**
Heart rate	150.96 (15.00)	151.56 (13.55)	0.897	147.11 (14.57)	171.74 (11.89)	**<0.0001**	146.42 (14.11)	152.95 (13.48)	0.153

*Note*: *p*‐values < 0.05 are presented in bold type.

Analysis of within‐group variability in unswaddled infants shows that there is an increase of vital signs and ALPS‐Neo score during weighing, whereas there is a decrease of all variables during post‐weight care (see Table 3). When infants were swaddled, vital signs and ALPS‐Neo scores decreased from basal care to weighing care, and remained stable from weighing care to post‐weight care.

**TABLE 3:** Within Group variability. Follow‐up of stress score and vital signs from pre‐weight care, to weight measurement and to post‐weight care. Quantitative data are presented as mean value (standard deviation).

	Pre‐weight care	Weight measurement	*p* Value	Weight measurement	Post‐weight care	*p* Value
Swaddy intervention						
ALPS‐Neo score	1.65 (1.45)	0.23 (0.42)	**0.0002**	0.23 (0.42)	0.05 (0.22)	0.102
Respiratory rate	58.10 (9.58)	46.10 (14.10)	**0.0041**	46.10 (14.10)	42.32 (9.38)	0.335
Heart rate	150.96 (15.00)	147.10 (14.57)	0.426	147.10 (14.57)	146.42 (14.11)	0.883
Control						
NEO score	1.26 (1.28)	4.97 (1.92)	**<0.0001**	4.97 (1.92)	1.65 (1.59)	**<0.0001**
Respiratory rate	46.91 (8.54)	66.47 (11.49)	**<0.0001**	66.47 (11.49)	54.84 (11.57)	**0.0036**
Heart rate	151.56 (13.55)	171.73 (11.89)	**<0.0001**	171.73 (11.89)	152.95 (13.48)	**<0.0001**

*Note*: *p*‐values < 0.05 are presented in bold type.

## 4 DISCUSSION

In this study, we explored whether swaddled preterm infants show less physiologic distress on intervention versus control days. Our study shows a significant reduction of RR, HR, and stress scores in swaddling groups when compared to no‐swaddling groups.

Our results are in line with a study dating back more than 20 years, in which preterm infants were exposed to both swaddled and unswaddled weighing (Neu & Browne, 1997). In that crossover design, each infant was weighed one night unswaddled and one night swaddled. There was a sharp increase in all scores (self‐regulatory activity, motor and physiologic organization) during unswaddled weighing. Conversely, when infants were swaddled during weighing procedures, all scores remained stable or even decreased; after weighing, scores increased slightly when the supportive swaddling was removed. As far as we know, no other research has addressed this topic. Our results are in agreement with the results for babies in the control group. Additionally, we report that lower RR and stress scores with *SWADDY* remain during post‐weight care. These results may indicate that *SWADDY*‐interventions provided more than short‐term relaxation for these preterm infants.

On the other hand, the potential benefits of bathing swaddled children have been studied in detail. A recent randomized trial reports that the stress score and the time spent crying of the swaddled bathing group, were less than in the control group after bathing (Huang et al., 2022). Similarly, another randomized trial that compared the effects of traditional tube bathing with swaddled bathing methods in 80 preterm newborns, showed that swaddled bathing appears to decrease stress symptoms and to stabilize HR within basal limits (Çaka & Gözen, 2018). Consistent with these results, another study has determined that infants' RR, HR, and pain scores were higher in the sponge bath condition than in the swaddle bathing group (Ceylan & Bolւşւk, 2018). With regard to the effects of non‐pharmacologic pain management, a study on BCG vaccination of healthy neonates, indicates that swaddling these babies within 4 min of the injection decreased their behavioural responses of pain and accelerated HR (Hashemi et al., 2016). Extensive research has been carried out since the 1980's to check whether or not swaddling alleviates pain‐elicited distress during the heelstick and other painful procedures in infants (Riddell et al., 2015). To summarize, pain intensity was severe in the non‐swaddling group and moderate in the swaddling group (Ho et al., 2016; Sleuwen et al., 2006). Finally, but equally important in order to further safeguard child development, a number of studies from recent years verify that preterm infants who are swaddled are less awake and have more sleep time (Abdeyazdan et al., 2016) In addition, since swaddling is a useful technique to induce sleep when performing magnetic resonance imaging, it avoids the use of anaesthesia with its associated risks (Antonov et al., 2017; Esmonde et al., 2019; Templeton et al., 2020).

Nonetheless, to get the full picture of outcomes of swaddling infants, previous reports that have warned against swaddling in some conditions cannot be ignored. Swaddling infants with the legs in extension and adduction may increase the risk of hip dysplasia. The combination of swaddling with prone position increases the risk of sudden infant death syndrome if infants attempt to turn. These adverse effects apply to full term neonates and not to preterm babies under intensive care (Nelson, 2017).

### 4.1 Limitations

Nonblinded observer bias occurs from the interaction of the subjectivity of the outcome and the predisposition of the observer. Predispositions vary significantly from trial to trial and from one observer to another. Conscientious nonblinded assessors may paradoxically produce a bias favouring the control in some trials by overcompensating for a predicted bias in favour of the experimental intervention. In contrast, fairly neutral assessors in other trials will have no significant bias. We do not know whether there is observer bias in this study, and whether this bias is one way or the other. This limitation applies to subjective measurements, but we have also obtained differences in objective measurements that are monitored, such as HR and RR, so they are not dependent on the observer.

Our study's weaknesses include the limited sample size and its observational nature. Our small sample size prevented us from detecting any differences in adjusted outcomes. In studies in which data are collected by more than one observer, kappa interobserver coefficients should be at least 0.70 (Cerda & Villarroel, 2008). In this analysis, inter‐rater reliability was >0.6 for 70% of measurements when assessing APLS‐Neo score. Also of note is the limited accuracy of clinically measured vital signs in our study. However, it has been shown that clinical measurements (by NICU nurses, as part of their usual practice) were not significantly different from standardized research measurements in different settings (Olsen et al., 2009). The primary use of our data is to support the establishment of a new weighing care program, and to generate baseline data for future research. Limitations in resources may challenge the feasibility of swaddling routine clinical care. This study has not addressed nurses' attitude or burden of work with regard to this new swaddling device. Future studies are needed to determine the acceptability of the intervention, through in‐depth interviews.

## 5 CONCLUSIONS

This study shows that a new swaddling device can contribute to maintaining stable vital signs, and may decrease stress experienced during weighing procedures, among preterm infants. Therefore, it is recommended to take advantage of these methods to optimize evidence‐based developmentally sensitive care in the NICU.

## 6 HOW MIGHT THIS INFORMATION AFFECT NURSING PRACTICE?

Nurses have a powerful role in controlling NICU environmental factors, responsible for negative effects on the neurodevelopmental outcomes of preterm infants. Neonatal nurses are primary points of contact for preterm infants receiving health care, providing them with the unique opportunity to decrease stress experienced by their patients during invasive procedures. Reduced motor activity during weight measurements has been found to stabilize infant heart rate, respiratory rate, and behaviour organization. Knowledge gained by identifying the benefits of swaddling and gentle touch for cardio‐respiratory stability can be transformed into routine nursing care within the NICU. Results from this study can be used by neonatal nurses to conduct weighing care with swaddling devices as an alternative, particularly when facilitated tucking is not feasible.

## AUTHOR CONTRIBUTIONS

SV‐P designed the study, collected the data and wrote the manuscript. GR performed the literature search and the modified discussion. IG performed the statistical analysis. TN was involved in monitoring the data upon which this manuscript is based. JP‐T was involved in the editing of the manuscript. SV finalized the manuscript. GG designed the study and critically analysed the results. All authors read and approved the final manuscript.

## FUNDING INFORMATION

This research was partly supported by the Nurse Research Projects (Grant number PR‐025/16) from the Nurse and Society Foundation (Barcelona, Spain) to the first author.

## CONFLICT OF INTEREST STATEMENT

All authors declare no actual or potential conflicts of interests.

## ETHICAL APPROVAL

This study was conducted with the approval of the Clinical Research Ethics Committee, Sant Pau Hospital (Barcelona). The authors certify that they have obtained all appropriate patient consent forms.

## Data Availability

The data that support the findings of this study are available on request from the corresponding author. The data are not publicly available due to privacy or ethical restrictions.
